# Association between inflammatory biomarker profiles and cardiovascular risk in individuals with and without HIV

**DOI:** 10.1097/QAD.0000000000003462

**Published:** 2022-12-14

**Authors:** Luxsena Sukumaran, Ken M. Kunisaki, Nicholas Bakewell, Alan Winston, Patrick W.G. Mallon, Nicki Doyle, Jane Anderson, Marta Boffito, Lewis Haddow, Frank A. Post, Jaime H. Vera, Memory Sachikonye, Caroline A. Sabin

**Affiliations:** a Institute for Global Health, University College London; b National Institute for Health Research (NIHR) Health Protection Research Unit (HPRU) in Blood-borne and Sexually Transmitted Infections at University College London, UK; c Minneapolis Veterans Affairs Healthcare System, University of Minnesota, Minneapolis, Minnesota, USA; d Department of Infectious Disease, Imperial College London, London, UK; e School of Medicine, University College Dublin, Dublin, Ireland; f Homerton University Hospital; g Chelsea and Westminster Healthcare NHS Foundation Trust; h Kingston Hospital NHS Foundation Trust; i King's College Hospital NHS Foundation Trust, London; j Brighton and Sussex Medical School, Brighton; k UK Community Advisory Board (UK-CAB), London, UK.

**Keywords:** biomarkers, cardiovascular disease, HIV, inflammation

## Abstract

**Background:**

People with HIV have an increased risk for cardiovascular morbidity and mortality. Inflammation and immune activation may contribute to this excess risk.

**Methods:**

We assessed thirty-one biomarkers in a subset of POPPY participants and identified three distinct inflammatory profiles: ‘gut/immune activation’, ‘neurovascular’, and ‘reference’ (relatively low levels of inflammation). Ten-year cardiovascular disease (CVD) risk predictions were calculated using the QRISK, Framingham Risk Score (FRS) and the Data Collection on Adverse effects of anti-HIV Drugs (D:A:D) algorithms. The distributions of CVD risk scores across the different inflammatory profiles, stratified by HIV status, were compared using median quantile regression.

**Results:**

Of the 312 participants included [70% living with HIV, median (interquartile range; IQR) age 55 (51–60) years; 82% male; 91% white], 36, 130, and 146 were in the ‘gut/immune activation’, ‘neurovascular’, and ‘reference’ cluster, respectively. The median (IQR) QRISK scores were 9.3% (4.5–14.5) and 10.2% (5.5–16.9) for people with and without HV, respectively, with similar scores obtained with the FRS and D:A:D. We observed statistically significant differences between the distributions of scores in the three clusters among people with HV. In particular, median QRISK [5.8% (1.0–10.7) and 3.1% (0.3–5.8)] scores were higher, respectively, for those in the ‘gut/immune activation’ and ‘neurovascular’ clusters compared to those in the reference cluster.

**Conclusions:**

People with HIV with increased gut/immune activation have a higher CVD risk compared to those with relatively low inflammation. Our findings highlight that clinically important inflammatory subgroups could be useful to differentiate risk and maximise prediction of CVD among people with HIV.

## Introduction

Increased access to antiretroviral therapy (ART) has significantly reduced HIV-associated morbidity and mortality [1,2]. Although HIV is now a manageable long-term condition, concerns have shifted to non-AIDS-related complications which may compromise the overall health of people living with HIV. Among these complications, cardiovascular disease (CVD) is a major cause of morbidity and mortality in people with HIV, including those with suppressed HIV RNA [3–7]. A recent large UK analysis of primary care data found that people with HIV had a 50% higher risk of a composite CVD outcome which comprised myocardial infarction (MI), stroke, ischemic heart disease, heart failure, and peripheral vascular disease, compared with the general population of similar age, gender, ethnicity, and geographic location [8]. The cause of this increased risk is likely multifactorial and extends beyond people with HIV having a higher prevalence of traditional CVD risk factors such as smoking, diabetes mellitus, and obesity [9]. Unique drivers of CVD risk hypothesized to play a role include HIV associated immune dysfunction and inflammation [10,11]. This is supported by several studies that have reported elevated biomarkers of inflammation, thrombosis, apoptosis, and myocardial injury in people with HIV compared to HIV-negative controls [12–14]. Several of these markers were also individually associated with cardiac dysfunction, independent of traditional and HIV-related risk factors [12].

Over the past decade, there have been increasing efforts to develop and evaluate CVD risk prediction tools to reduce incidence of CVD and aid clinical management in HIV populations. General population-derived algorithms include QRISK [15], which is recommended by The National Institute for Health and Care Excellence (NICE) for UK research and clinical practice, and the Framingham CVD Risk Score (FRS) [16], which was developed using data from the United States-based Framingham Heart Study. However, neither the FRS nor QRISK consider independent HIV-related risk factors that may drive CVD risk in an HIV population. In an attempt to improve CVD predictions in people with HIV, the Data Collection on Adverse Events of Anti-HIV Drugs (D:A:D) risk score was developed, using data from a large cohort of predominantly European people living with HIV (*N* > 30 000), and incorporates information on CD4 lymphocyte count, exposure to protease inhibitors (PIs), and current use of abacavir, as well as traditional CVD risk factors. It is important to note that several studies have suggested that specific protease inhibitors (PIs), particularly those associated with PI-boosted regimens incorporating ritonavir, may directly increase proinflammatory signalling (regardless of concomitant HIV infection) [17,18]; highlighting the importance of their inclusion in the algorithm. Despite this, established CVD risk models may not accurately estimate CVD risk among people with HIV as they do not consider the relative contributions of HIV-associated inflammation and immune dysregulation. Incorporating such biomarker data could improve CVD risk stratification, elucidate underlying immunologic abnormalities associated with CVD risk in people with HIV, and guide clinical decision making.

In previous work, we used data from the Pharmacokinetic and clinical Observations in People over Fifty (POPPY)-Sleep sub-study to generate clinically relevant individual subgroups based on 31 plasma protein biomarkers [19]. Building on this work, the aim of the current study was to evaluate the associations between the inflammatory profiles and 10-year CVD risk, predicted using the QRISK, FRS, and D:A:D equations, in people with HIV and demographically/lifestyle-similar people without HIV.

## Methods

### Study population

The POPPY study is a prospective observational study, initiated in 2013, to examine the clinical outcomes of people with HIV from seven clinics in the UK, and one in Ireland. Characteristics and eligibility criteria of the POPPY cohort are detailed elsewhere [20]. Briefly, POPPY includes three cohorts: people with HIV aged ≥50 years (older people with HIV), people with HIV aged 18–49 years (younger people with HIV), and HIV-negative controls aged ≥50 years who were frequency-matched to the older people with HIV on gender, ethnicity, sexual orientation, and location (in or out of London). Participants were recruited from April 2013 to January 2016. Information on socio-demographic characteristics, established CVD risk factors, prescribed co-medications, comorbidities, and laboratory measurements were collected at study visits by trained clinical staff, and through data linkage with the UK Collaborative HIV Cohort (UK CHIC) study and the Mater Misericordiae University Hospital (MMUH) Infectious Diseases (ID) Cohort in Dublin.

A subset of 483 POPPY participants, selected independently of any existing sleep symptoms, were recruited into the POPPY-Sleep sub-study [21], if they were able/willing to wear a fingertip oximetry device and wrist actigraph for a week and based on the investigator's judgement about whether participants could adhere to study procedures. Of these, 465 had reliable biomarker measurements (collected at or near enrolment between March 2017 and July 2018) and were subsequently included in the analysis to identify inflammatory profiles. For the present study, participants were additionally required to have complete data for the calculation of the selected CVD risk algorithms and for the covariates included in the adjusted regression models. We restricted our analyses to those aged 40–75 years (*n* = 4 excluded) to ensure consistency and validity with the risk algorithms. Individuals who reported prior CVD at baseline were not excluded as the number of people affected was very small (Table 1, Supplemental Digital Content).

All participants provided written informed consent and ethical approval was granted by the UK National Research Ethics Service (NRES; Fulham London, UK number 12/LO/1409) for the POPPY study and the UK Health Research Authority & Research Ethics Committee (number 16/LO/2175) for the POPPY-Sleep sub-study.

### Cardiovascular disease risk prediction algorithms

The primary outcome of this study was 10-year CVD risk, which was determined using each of three validated algorithms: the QRISK, FRS, and D:A:D score (with the latter calculated only for those with HIV). These algorithms were selected since they are recommended by the UK (QRISK) and European (FRS and D:A:D) HIV treatment guidelines. An adapted version of the QRISK2 equation, that assumed no variation in risk scores across the UK (i.e. that excluded information on postcode), was used as we did not collect individual postcodes. However, similar to previous work [22], a sensitivity analysis was conducted using the participant's hospital postcode as a proxy (excluding participants from the Dublin cohort with no postcode data available). Although the D:A:D score was originally developed to estimate 5-year CVD risk, we used the 10-year risk equation [23] to ensure consistency with the other included algorithms.

### Variables and covariates

A comparison of the CVD risk factors and predictors used by the three CVD risk models are described in Table 2, Supplemental Digital Content. Smoking status was ascertained from the self-reported questionnaires completed at the POPPY visit. Conditions associated with cardiovascular risk (diabetes and hypertension, rheumatoid arthritis, kidney disease, and atrial fibrillation) were defined as: use of relevant medication, and/or a self-reported diagnosis. Participants with no self-reported records of family history of CVD were subsequently coded as having no family history of CVD. HIV-related information for the D:A:D equation, that is, use of ART drugs (cumulative PI and/or nucleoside reverse transcriptase inhibitor (NRTI) use and current abacavir use) and CD4^+^ cell count were obtained from linkage with the UK CHIC and the Dublin ID cohort. In addition to the components of the CVD risk prediction algorithms, we also accessed data on statin use, current and nadir CD4^+^ T-cell counts, and plasma HIV RNA load (undetectable viral load defined as ≤50 copies/mL).

### Statistical analysis

The distribution of demographic characteristics, CVD risk factors, and HIV-related factors are presented as counts (percentages) for categorical variables and medians (interquartile ranges [IQRs]) for quantitative variables. The analysis presented are based on inflammatory profiles (generated using data from both people with HIV as well as HIV-negative controls) identified from a previous study [19]. These profiles were generated using thirty-one biomarkers, related to eight inflammatory pathways (Table 3, Supplemental Digital Content), that were analysed at the Centre for Experimental Pathogen Host Research (CEPHR), University College Dublin (UCD) using two immunoassay platforms based on Enzyme Linked Immunosorbent Assay (ELISA); Meso Scale Discovery (MSD; Rockland, Maryland, USA) and Luminex − MAGPIX (Luminex; R&D Systems, Minneapolis, Minnesota, USA). As previously published, principal component analysis (PCA) followed by unsupervised agglomerative hierarchical cluster analysis identified three distinct inflammatory profiles: a ‘gut/immune activation’ cluster (upregulation in cytokines and biomarkers associated with gut microbial translocation); a ‘neurovascular’ cluster (upregulation in vascular, neuronal and coagulation-associated markers); and a final cluster (no distinct upregulation in any included biomarkers associated with inflammatory pathways) that we have designated as the ‘reference cluster’. The association between these profiles and CVD risk (predicted using QRISK, FRS and D:A:D score) was assessed using a series of median quantile regression models. Confounder variables included in the adjusted models were identified *a priori* and differed for each CVD risk algorithm, reflecting the fact that each algorithm incorporated a slightly different set of factors. All three risk models adjusted for statin use. Additional confounders adjusted in the FRS and D:A:D models were ethnicity and BMI (already incorporated in QRISK algorithm). The D:A:D model (calculated only in the subgroup with HIV) allowed us to adjust for additional HIV-related factors including time since HIV diagnosis, nadir CD4^+^ cell count, years of ART use, and plasma HIV RNA load. Furthermore, the FRS and QRISK models were stratified by HIV status to identify whether the associations of inflammatory profiles with predicted CVD risk differed between those with and without HIV. Missing data were handled using listwise deletion, excluding any participants with missing data on any variable. Three sensitivity analyses were conducted (Tables 4–6, Supplemental Digital Content): QRISK analysis using hospital postcodes as a proxy, analysis excluding participants with a prior CVD event at baseline, and analysis excluding statin use as a confounder as the nature of our analysis (cross-sectional) could not ascertain whether statins were used as a result of a prior CVD event or in a preventive way. For the purpose of these analyses, the two cohorts of older and younger people with HIV were combined to allow HIV status and age to be considered as individual factors. All analyses were performed using Stata, version 17 (StataCorp, College Station, Texas, USA). A two-sided *P*-value ≤0.05 was considered statistically significant for all analyses.

## Results

### Participant characteristics

Of the 465 participants, 153 (33%) were excluded to ensure QRISK, FRS, and D:A:D scores were all measured in the same population (i.e., participants were excluded if they did not have complete data for the predictors included in any of the three algorithms), leaving 312 participants, 218 of whom were living with HIV. The population excluded reported similar baseline characteristics to those included (Table 7, Supplemental Digital Content). The baseline demographic and clinical characteristics of those included are detailed in Table 1, stratified by HIV status and cluster. Included participants had a median age of 55 years (IQR 51–60), were predominately male (82.3%) and of white ethnicity (91.4%). With respect to the traditional CVD risk factors, 24.4% of the total cohorts were current smokers, 20.8% had diabetes, and the median (IQR) BMI was 25.6 kg/m^2^ (23.1–28.4). The median (IQR) systolic blood pressure was 126 mmHg (116–140) and 58 participants (19.5%) were reported to be taking statins. Of the HIV parameters, the median years since HIV diagnosis was 16.1 (8.2–21.9), the median CD4^+^cell count was 607 cells/μl (468–756), 93.6% had an undetectable viral load, and the median duration of ART use was 10.3 years (5.4–17.1).

**Table 1:** Demographic and clinical characteristics stratified by HIV status and cluster.

	Population			Cluster		
Characteristic *n* (%) or median (IQR)	Whole study population (*n* = 312)	People without HIV (*n* = 94)	People with HIV (*n* = 218)	Reference (*n* = 146)	Gut/immune activation (*n* = 36)	Neurovascular (*n* = 130)
*Cluster*						
Reference	146 (46.8)	47 (50.0)	99 (45.4)	–	–	–
Gut/immune activation	36 (11.5)	17 (18.1)	19 (8.7)	–	–	–
Neurovascular	130 (41.7)	30 (31.9)	100 (45.9)	–	–	–
*Demographic*						
Age, years	55 (51–60)	58 (54–61)	54 (50–59)	55 (50–60)	57 (52–62)	55 (51–60)
Male	258 (82.3)	62 (66.0)	196 (89.9)	119 (81.5)	27 (75)	112 (86.2)
White	285 (91.4)	285 (91.4)	198 (90.8)	135 (92.5)	32 (88.9)	118 (90.8)
*Cardiovascular risk factors*						
Statin use	58 (18.6)	12 (12.8)	46 (21.1)	27 (18.5)	3 (8.3)	28 (21.5)
Diabetes mellitus	65 (20.8)	21 (22.3)	44 (20.2)	31 (21.2)	10 (27.8)	24 (18.5)
Systolic blood pressure, mmHg	126 (116–140)	130 (116–142)	126 (116–137)	126 (116–136)	134 (125–154)	126 (115–140)
Total cholesterol mmol/l	5 (4.3–5.7)	5.4 (4.6–6.0)	4.9 (4.2–5.5)	5 (4.3–5.7)	4.9 (4.2–5.6)	5.1 (4.4–5.7)
HDL cholesterol mmol/l	1.3 (1.1–1.6)	1.4 (1.2–1.7)	1.3 (1.0–1.5)	1.3 (1.1–1.6)	1.4 (1.1–1.6)	1.3 (1.1–1.5)
Body mass index kg/m^2^	25.6 (23.1–28.4)	26.2 (24.1–29.2)	25.1 (22.9–28.2)	24.8 (22.5–27.3)	25.8 (24–29.1)	26.4 (23.7–29.7)
Smoking						
Never	130 (41.7)	45 (47.9)	85 (39.0)	62 (42.5)	17 (47.2)	51 (39.2)
Former	106 (34.0)	34 (36.2)	72 (33.0)	57 (39.0)	10 (27.8)	39 (30.0)
Current	76 (24.4)	15 (16.0)	61 (28.0)	27 (18.5)	9 (25.0)	40 (30.8)

*HIV-related factors*				Reference (*n* = 99)	Gut/immune activation (*n* = 19)	Neurovascular (*n* = 100)
Years since HIV diagnosis	-	-	16.1 (8.2−21.9)	13.9 (7.7−21.7)	13.0 (8.2−18.0)	17.3 (9.6−22.6)
CD4^+^ cell count, cells/μl	-	-	607.5 (468−756)	597 (472−760)	656 (460−792)	606 (458−750)
Nadir CD4^+^ cell count, cells/μl	-	-	180 (99−280)	218 (120−302)	160 (108– 250)	143 (75−250)
HIV-RNA undetectable (<50 copies/ml)	-	-	204 (93.6)	95 (96.0)	16 (84.2)	93 (93.0)
ART duration, years	-	-	10.3 (5.4−17.1)	8.5 (4.4−15.6)	12.6 (7.1−16.7)	11.7 (5.9−17.6)
Current abacavir use	-	-	29 (13.3)	13 (8.9)	2 (5.5)	14 (10.8)
Any PI use	-	-	99 (45.4)	42 (42.4)	11 (57.9)	46 (46.0)
Cumulative PI use	-	-	2.31 (0.0−7.8)	1.91 (0.0–7.5)	3.7 (0.0–8.7)	2.3 (0.0–7.9)
Any NRTI use	-	-	190 (87.2)	88 (88.9)	16 (84.2)	86 (86.0)
Cumulative NRTI use	-	-	9.0 (3.7–13.9)	6.8 (3.0–13.2)	9.8 (5.0–13.4)	10.2 (4.4–14.8)

ART, antiretroviral therapy; IQR, interquartile range; NRTI, nucleoside reverse transcriptase inhibitor; PI, protease inhibitor.

### Cardiovascular disease risk across inflammatory profiles

#### Whole study population

The median (IQR) 10-year CVD risk scores using FRS and QRISK were 11.8% (6.8–18.7) and 9.5% (5.0–15.7), respectively (Table 2). The number of participants categorized as being at moderate (10–19%) or high (≥20%) CVD risk, respectively, were 119 (38.1%), and 66 (21.2%) using the FRS score and 115 (36.9%) and 35 (11.2%) using QRISK. Both the median (IQR) FRS and QRISK scores were higher among those in the ‘gut/immune activation’ [FRS: 13.5% (8.1–25.4); QRISK: 13.0% (4.7–17.4) and ‘neurovascular’ cluster [FRS: 13.6% (8.4–19.6); QRISK: 10.7% (5.9–16.2)] compared to those in the ‘reference’ cluster [FRS: 10.2 (5.9–16.0); QRISK: 7.8% (4.1–12.8)].

**Table 2:** CVD risk by HIV status and inflammatory biomarker profiles.

	Population			Cluster		
CVD risk algorithm	Whole study population (*n* = 312)	People without HIV (*n* = 94)	People with HIV (*n* = 218)	Reference (*n* = 146)	Gut/immune activation (*n* = 36)	Neurovascular (*n* = 130)
*QRISK*						
Median (IQR)	9.5 (5.0−15.7)	10.2 (5.5−16.9)	9.3 (4.5−14.7)	7.8 (4.1–12.8)	13.0 (4.7−17.4)	10.7 (5.9−16.2)
Level of risk						
Low (<10%)	162 (51.9)	45 (47.9)	117 (53.7)	89 (61.0)	16 (44.4)	57 (43.9)
Moderate (10–20%)	115 (36.9)	35 (37.2)	80 (36.7)	43 (29.5)	15 (41.7)	57 (43.9)
High (≥20%)	35 (11.2)	14 (14.9)	21 (9.6)	14 (9.6)	5 (13.9)	16 (12.3)
*FRS*						
Median (IQR)	11.8 (6.8–18.7)	11.0 (7.3−19.6)	12.1 (6.8−18.4)	10.2 (5.9−16.0)	13.5 (8.2−25.4)	13.6 (8.4−19.9)
Level of risk						
Low (<10%)	127 (40.7)	44 (46.8)	83 (38.1)	72 (49.3)	12 (33.3)	43 (33.1)
Moderate (10–20%)	119 (38.1)	29 (30.9)	90 (41.3)	54 (37.0)	10 (27.8)	55 (42.3)
High (≥20%)	66 (21.2)	21 (22.3)	45 (20.6)	20 (13.7)	14 (38.9)	32 (24.6)

	Whole population (*n* = 218)	People without HIV (*n* = 94)	People with HIV (*n* = 218)	Reference (*n* = 99)	Gut/immune activation (*n* = 19)	Neurovascular (*n* = 100)
*D:A:D*						
Median (IQR)	–	–	9.0 (5.0−14.7)	7.3 (4.3−12.4)	14.2 (7.1−19.7)	9.8 (5.8−16.0)
Level of risk	–	–				
Low (<10%)	–	–	125 (57.3)	66 (66.7)	7 (36.8)	52 (52.0)
Moderate (10–20%)	–	–	68 (31.2)	24 (24.2)	8 (42.1)	36 (36.0)
High (≥20%)	–	–	25 (11.5)	9 (9.1)	4 (21.1)	12 (12.0)

CVD, cardiovascular disease; D:A:D, Data Collection on Adverse effects of anti-HIV Drugs; FRS, Framingham Risk Score.

#### People with HIV subgroup

The D:A:D algorithm presented a median (IQR) 10-year risk score of 9.0% (5.0–14.7), with 68 (31.2%) and 25 (11.5%) participants identified as having moderate and high CVD risk, respectively (Table 2). A larger median (IQR) risk was observed among people with HIV in the ‘gut/immune activation’ cluster [14.2% (7.0–19.7)] than those in the ‘neurovascular’ cluster [9.8% (5.8–16.0)], when compared with the ‘reference’ cluster [7.3% (4.3–12.4)].

### Association of Framingham Risk Score and QRISK with inflammatory profiles by HIV status

**Fig. 1:**
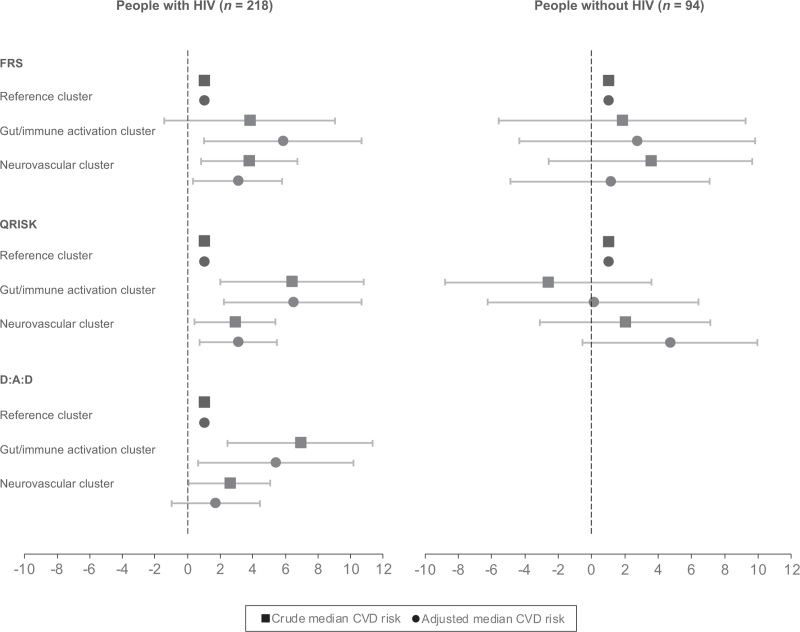
Differences in the median CVD risk scores across inflammatory profiles relative to the reference cluster.

People with HIV in the ‘gut/immune activation’ and ‘neurovascular’ cluster demonstrated a higher median FRS and QRISK score, compared to their HIV-negative counterparts, in both the unadjusted and adjusted models (Fig. 1). Among the HIV-negative cohort, median FRS and QRISK scores were relatively higher for those in the ‘neurovascular’ cluster than the ‘gut/immune activation’ cluster, when compared to the ‘reference’ cluster. However, these associations were not statistically significant prior to or after adjustment for relevant covariates. In contrast, among people with HIV, the adjusted median scores, when compared with the ‘reference’ cluster, were significantly higher for those in the ‘gut/immune activation’ cluster, with a higher risk reported by QRISK {FRS: 5.8% [95% confidence interval (CI): 1.0–10.7]; QRISK: 6.5% (95% CI: 2.2–10.7)}, than the ‘neurovascular’ cluster [FRS: 3.1% (95% CI: 0.3–5.8); QRISK: 3.1% (95% CI: 0.7–5.5)].

95% confidence intervals are displayed. All three risk models adjusted for statin use. FRS and D:A:D also adjusted for ethnicity and BMI. D:A:D further adjusted for time since HIV diagnosis, nadir CD4^+^ cell count, years of ART use, and plasma HIV RNA load. CVD, cardiovascular disease; FRS, Framingham Risk Score; D:A:D, Data Collection on Adverse effects of anti-HIV Drugs.

### Association of Data Collection on Adverse effects of anti-HIV Drugs risk with inflammatory profiles among people with HIV

In the subgroup of people with HIV, median D:A:D scores were also higher for those in the ‘gut/immune activation’ cluster compared to the ‘neurovascular’ cluster (Fig. 1). The unadjusted median D:A:D scores [6.9% (95% CI: 2.5–11.4)] remained significantly higher for people with HIV in the ‘gut/immune activation’ cluster after adjustment for potential confounders [5.4% (95% CI: 0.7–10.2)]. However, this statistical significance was not retained for the median D:A:D score for people with HIV in the ‘neurovascular’ cluster, compared to those in the ‘reference’ cluster, after adjustment [unadjusted model: 2.6% (95% CI: 0.1–5.1), *P* = 0.05; adjusted model: 1.7% (95% CI: −1.0–4.4), *P* = 0.21].

### Sensitivity analyses

The first sensitivity analysis conducted applied postcode limits to the sample for QRISK calculation. We observed that including postcodes (hospital postcodes as a proxy) resulted in very similar CVD risk to that observed in the primary analyses (Table 4, Supplemental Digital Content). The second sensitivity analysis excluded participants that reported a prior CVD event at baseline (reduced sample size by 43%; *n* = 177). Similar results to the primary analysis were observed with CVD risk among HIV-negative controls and people with HIV when calculated using the FRS and QRISK algorithms (Table 5, Supplemental Digital Content). However, statistical significance observed in the association between D:A:D score and people with HIV in the ‘neurovascular’ cluster was lost. The final sensitivity analysis, where we excluded statin use as a confounder, also presented similar results to the primary analysis (Table 6, Supplemental Digital Content).

## Discussion

In this well characterized cohort of 218 people with HIV and 94 HIV-negative individuals, we found that inflammatory phenotypes, identified using biomarker-derived clusters, were associated with well established CVD risk prediction scores. All three CVD risk algorithms included in our study reported a higher median CVD risk for people with HIV in the ‘gut/immune activation’ cluster and the ‘neurovascular’ cluster, when compared to those in the ‘reference’ cluster. The magnitude and significance of most of these associations remained after controlling for potential confounders. In particular, median FRS and QRISK scores for people with HIV in the ‘gut/immune activation’ cluster were almost double that of those in the ‘neurovascular’ cluster, suggesting that, as anticipated, differences in distribution of CVD risk among people with HIV may be dependent on immunological factors. This also supports previous work that reported associations between gut microbial translocation markers and both CVD events and surrogate markers of CVD (e.g. carotid intimal/medial thickening) among people with HIV [24,25]. Thus, our work further emphasizes the potential importance of using biomarker data to improve our understanding on the role of inflammation and immune activation in CVD risk prediction in people living with HIV. Associations between traditional risk factors and the clusters were also observed in the present study. People with HIV in both the ‘gut/immune activation’ and ‘neurovascular’ clusters had a higher BMI compared to those in the ‘reference’ cluster. People with HIV in both these clusters also had lower nadir CD4^+^ cell counts consistent with having more advanced HIV disease. The former cluster was also characterized by older age and a higher prevalence of diabetes, while the latter had a higher proportion of current smokers and higher statin use, compared to the ‘reference’ cluster. A higher use of statins among individuals in the neurovascular cluster may explain the lower CVD risk in this group than among individuals in the gut/immune activation cluster. Further work is required to explore the relationship between statin use and CVD risk across the inflammatory profiles. Clustering techniques have been used by several previous studies to identify clinically relevant subgroups within other, similarly complex, conditions such as Parkinson's disease and COPD, improving understanding on their pathophysiology [26,27]. In particular, McGettrick *et al.* recently reported associations between inflammatory clusters (characterized by either T-cell senescence and exhaustion or systemic inflammation) and subclinical coronary artery disease in individuals with HIV [28]. However, to our knowledge this is the first study to explore associations between inflammatory phenotypes and existing CVD risk prediction models in a cohort of people with HIV. Although associations between single-biomarker models and CVD risk have been well documented among people with HIV [29–31], the associations between these biomarkers when considered together and CVD risk are unclear. To date, novel biomarkers have not been evaluated with regards to their ability to improve CVD risk stratification models among people with HIV. Our findings suggest that unique HIV-related factors such as immune dysregulation and inflammation may provide additional predictive information alongside traditional CVD risk factors when differentiating risk and maximizing prediction of CVD among people with HIV and inform appropriate implementation of preventive and therapeutic measures.

The main limitation of our study is that our analysis was cross-sectional, and therefore we did not have any information on incident CVD to examine the predictive performance of these CVD risk algorithms for future cardiovascular events across the different inflammatory profiles among people with HIV. Our results should also be interpreted with caution given the small sample sizes of the clusters, especially in that of the gut/immune activation cluster, thus further validation is needed from other cohorts. The inflammatory profiles were restricted to data on 31 biomarkers; although other biomarkers may have been involved with the profiles identified (e.g. lipopolysaccharide and bacterial rRNA and the gut/inflammation cluster), we deliberately chose to focus on host biomarker responses (e.g. SCD14 and i-FABP) [32], rather than markers of bacterial burden or translocation, as the latter are suggested to provide less reliable measurements due to susceptibility to contamination and inhibition by components of plasma [33]. Furthermore, our findings may not be generalizable to other HIV populations with different demographic characteristics, as our cohort was predominately men of white ethnicity. Analyses therefore could not include gender- or race-stratified results given the small number of female and non-white participants. Thus, larger, and more diverse, populations are essential to investigate whether our findings are comparable across other subgroups such as women and people of black African origin.

In summary, the findings presented highlight the need for more tailored and detailed descriptions of CVD risk among distinct subgroups of people with HIV, especially when considering that the difference in CVD risk – a common comorbidity among people with HIV – was statistically significant across the three inflammatory profiles identified. Additionally, we highlight that immunological pathways may be an important factor to consider alongside traditional CVD- and HIV-related risk factors in validated CVD risk algorithms. Future longitudinal studies are needed to assess temporal relationships and whether risk prediction models, accounting for immunological mechanisms, can improve predictions of future cardiovascular events among people with HIV.

## Acknowledgements

We thank all participants and staff involved in both the main POPPY study and the Sleep sub-study. POPPY Management Team: Marta Boffito, Paddy Mallon, Frank Post, Caroline Sabin, Memory Sachikonye, Alan Winston, Amalia Ndoutoumou, Daphne Babalis. POPPY Scientific Steering Committee: Jane Anderson, David Asboe, Marta Boffito, Lucy Garvey, Paddy Mallon, Frank Post, Anton Pozniak, Caroline Sabin, Memory Sachikonye, Jaime Vera, Ian Williams, Alan Winston. POPPY-Sleep Management Team: Ken Kunisaki, Susan Redline, Alan Winston, Caroline Sabin, Patrick Mallon, Nicki Doyle, and Amalia Ndoutoumou. POPPY methodology/statistics: Caroline Sabin, Nicholas Bakewell, Hajra, Okhai, Luxsena Sukumaran. POPPY-Sleep Sleep Reading Centre Team: Emily Kaplan, Dan Mobley, Michael Rueschman, and Michelle Reid (Brigham and Women's Hospital, Boston, USA). POPPY-Sleep methodology/statistics/analysis: Caroline Sabin and Nicholas Bakewell. POPPY Sites and Trials Unit: Caldecot Centre, King's College Hospital (Frank Post, Lucy Campbell, Selin Yurdakul, Sara Okumu, Louise Pollard, Beatriz Santana Suárez) Department of Infection and Population Health, UCL (Ian Williams, Damilola Otiko, Laura Phillips, Rosanna Laverick, Michelle Beynon, Anna-Lena Salz, Abigail Severn) Elton John Centre, Brighton and Sussex University Hospital (Martin Fisher, Amanda Clarke, Jaime Vera, Andrew Bexley, Celia Richardson, Sarah Kirk, Rebecca Gleig) Centre for Experimental Pathogen Host Research, School of Medicine, University College Dublin (Paddy Mallon, Alan Macken, Bijan Ghavani-Kia, Joanne Maher, Maria Byrne, Ailbhe Flaherty, Aoife McDermott, Riya Negi, Alejandro Garcia-Leon) Homerton Sexual Health Services, Homerton University Hospital (Jane Anderson, Sifiso Mguni, Rebecca Clark, Rhiannon Nevin-Dolan, Sambasivarao Pelluri) Ian Charleson Day Centre, Royal Free Hospital (Margaret Johnson, Nnenna Ngwu, Nargis Hemat, Anne Carroll, Sabine Kinloch, Mike Youle and Sara Madge) Imperial Clinical Trials Unit, Imperial College London (Daphne Babalis, Jodi Meyerowitz, Christina Prechtl) St. Mary's Hospital London, Imperial College Healthcare NHS Trust (Alan Winston, Lucy Garvey, Merle Henderson, Claire Peterson, Wilbert Ayap, Allan Lisenco) St Stephen's Centre, Chelsea and Westminster Hospital (Marta Boffito, David Asboe, Anton Pozniak, Margherita Bracchi, Nicole Pagani, Maddalena Cerrone, Daniel Bradshaw, Francesca Ferretti, Chris Higgs, Elisha Seah, Stephen Fletcher, Michelle Anthonipillai, Ashley Moyes, Katie Deats, Irtiza Syed, Clive Matthews, Peter Fernando). POPPY-Sleep Sites and Trials Unit: Caldecot Centre, King's College Hospital (Frank Post, Beatriz Santana Suarez, Lucy Campbell); Department of Infection and Population Health, University College London (Lewis Haddow, Michelle Beynon, Abigail Severn, Anna-Lena Salz, and Hinal Lukha); Elton John Centre, Brighton and Sussex University Hospital (Jaime Vera, Rebecca Gleig, and Sarah Kirk); Mater Misericordiae University Hospital (Aoife Cotter, Padraig McGettrick, Tara McGinty, Gerard Sheehan, Jack Lambert), Centre for Experimental Pathogen Host Research, School of Medicine, University College Dublin (Patrick Mallon, Alan Macken, Alejandro Garcia-Leon, Riya Negi, Dana Alawan); Homerton Sexual Health Services, Homerton University Hospital (Jane Anderson, Sambasivarao Pelluri, and Anna Price); St. Mary's Hospital London, Imperial College Healthcare NHS Trust (Alan Winston, Felix Dransfield); St Stephen's Centre, Chelsea and Westminster Hospital (Marta Boffito, Michelle Anthonipillai, Peter Fernando, Shane Hardwick, Chido Chiwome, Candida Fernandez, and Ana Milinkovic).

We acknowledge members of the NIHR HPRU Steering Committee: Professor Caroline Sabin (HPRU Director), Dr John Saunders (UKHSA Lead), Professor Catherine Mercer, Dr Hamish Mohammed, Professor Greta Rait, Dr Ruth Simmons, Professor William Rosenberg, Dr Tamyo Mbisa, Professor Rosalind Raine, Dr Sema Mandal, Dr Rosamund Yu, Dr Samreen Ijaz, Dr Fabiana Lorencatto, Dr Rachel Hunter, Dr Kirsty Foster and Dr Mamoona Tahir. The views expressed are those of the authors and not necessarily those of the NIHR, the Department of Health and Social Care, UKHSA, the US government, National Institutes of Health or Department of Veterans Affairs.

Financial disclosure: The POPPY-Sleep sub-study (which these analyses used biomarker data collected from) was funded by National Heart Lung and Blood Institute (R01 HL131049). The parent POPPY Study is primarily funded by investigator-initiated grants from BMS, Gilead Sciences, Janssen, MSD and ViiV Healthcare. We acknowledge the use of the National Institute for Health Research (NIHR)/Wellcome Trust Clinical Research Facility at King's College Hospital. The research is also funded by the National Institute for Health Research Biomedical Research Centre based at Imperial College Healthcare NHS Trust and Imperial College London. This material is also the result of work supported with resources and the use of facilities at the Minneapolis Veterans Affairs Medical Center, Minneapolis, USA. LS was funded through the National Institute for Health and Care Research Health Protection Research Unit (NIHR HPRU, Grant no.: NIHR200911) in Blood Borne and Sexually Transmitted Infections at University College London in partnership with the UK Health Security Agency (UKHSA). The funders had no role in the design of this study, analyses, interpretation of the data, or decision to submit results.

### Conflicts of interest

C.S. reports receipt of funding from Gilead Sciences and ViiV Healthcare for membership of Advisory Boards and for preparation of educational materials. K.M.K. has received consultancy fees from Allergan/AbbVie and Data and Safety Monitoring Board activity fees from Nuvaira and Organicell, outside the work presented here. A.W. has received speaker fees, advisory board honoraria or grants via Imperial College London from Gilead Sciences, ViiV Healthcare, MSD and Janssen. P.W.G.M. has received honoraria and/or travel grants from Gilead Sciences, MSD, Bristol-Myers Squibb, and ViiV Healthcare, and has been awarded grants by Science Foundation Ireland, outside the submitted work. J.A. reports personal fees from Gilead Sciences and ViiV; all outside of the work reported here. M.B. has acted as a speaker or adviser to, has been an investigator for, or has received grants to her institution from Gilead, ViiV, Janssen, B.M.S., Teva, Cipla, Mylan, and MSD; all outside the work presented here. F.A.P. reports grants and/or personal fees from Gilead Sciences, ViiV, Janssen, and MSD; all outside of the work reported here. J. V. reports travel, research grants, and personal fees from Merck, Janssen Cilag, Piramal Imaging, ViiV Healthcare, and Gilead sciences; all outside of the work reported here.

## Supplementary Material

Supplemental Digital Content
