# Supplementary material for: Association between inflammatory biomarker profiles and cardiovascular risk in individuals with and without HIV
Source: AIDS. 2022 Dec 14;37(4):595–603. doi: 10.1097/QAD.0000000000003462 (PMC9994838; doi:10.1097/QAD.0000000000003462)
Supplement: Supplemental Digital Content [file aids-37-595-s001.docx]

Supplementary appendix

**Supplementary Table 1.** Number of individuals who reported prior CVD at baseline

| **Cardiovascular event*** | **Total  (n=312)** | **People without HIV (n=94)** | **People with HIV (n=218)** |
| --- | --- | --- | --- |
| Angina | 5 (1.6) | 1 (1.1) | 4 (1.8) |
| Narrowed blood vessels | 5 (1.6) | 1 (1.1) | 4 (1.8) |
| Heart failure | 1 (0.3) | 0 (0.0) | 1 (0.5) |
| Hypertension | 65 (20.8) | 17 (18.1) | 48 (22.0) |
| Coronary artery bypass graft | 5 (1.6) | 2 (2.1) | 3 (1.4) |
| Dyslipidemia | 98 (31.4) | 23 (24.5) | 75 (34.4) |
| Arrhythmia | 1 (0.3) | 0 (0.0) | 1 (0.5) |
| CVD other | 16 (5.1) | 3 (3.2) | 13 (6.0) |
| ** Myocardial infarction, ischemic heart disease, transient ischemic attack, and atrial fibrillation were not reported by any of the participants included in this analysis at baseline.* | | | |
|  |  |  |  |

**Supplementary Table 2.** A comparison of the CVD risk factors and predictors used by the three CVD risk prediction models

| **Inflammatory pathway** | **Biomarkers analysed** |
| --- | --- |
| Atherosclerosis | MPO, IpPla-2 |
| Coagulation | D-Dimer, P-Selectin, CD 40 |
| Endothelial function | vWF, E-Selectin, VCAM1, ICAM-1 |
| Innate immune activation | CD14, IL-10, MCP-1, CD163, MP-Alpha1 |
| Microbial translocation | LBP, IL-18, IL-12, IFABP |
| Systemic inflammation | CRP, IFN-gamma, TNF-alpha, IL-6, IL-2, IL-1 Beta, TNF RII, TNF RI |
| Immune regulation | PDL-1, IL-4, IL-1RA |
| Axonal injury | NFL, S100B |
| *Reference cluster*; relatively low levels of all the included biomarkers  *Gut/immune activation cluster*; relatively higher levels of the microbial translocation (IL-12, IFABP), immune activation (IL-10), systematic inflammation (IFN-γ, TNF-α, IL-6, IL-2, IL-1β) and immune regulation (IL-4) markers  *Neurovascular cluster*; relatively higher levels of the coagulation, vascular and neuronal markers | |

**Supplementary Table 3.** A list of the thirty-one biomarkers (from 8 inflammatory pathways) used to generate the three distinct inflammatory clusters

**Supplementary Table 4.** Sensitivity analysis that included postcode data (hospital postcodes as a proxy) in QRISK calculation

| Sensitivity analysis 1 - QRISK | | | | | | | | | | |
| --- | --- | --- | --- | --- | --- | --- | --- | --- | --- | --- |
|  |  | **HIV-negative cohort** | | | |  | **HIV- positive cohort** | | | |
|  |  | **Crude (n=86)** | | **Adjusted (n=86)** | |  | **Crude (n=207)** | | **Adjusted (n=207)** | |
|  |  | Estimated impact on median | p-value | Estimated impact on median | p-value |  | Estimated impact on median | p-value | Estimated impact on median | p-value |
| Model 1: with  postcode data | *Reference*  *(n=42)* | *Ref* | *-* | *-* | *-* | *Reference*  *(n=96)* | *Ref* | *-* | *-* | *-* |
|  | Gut/immune activation  (n=17) | -2.7 (-9.2 – 3.8) | 0.41 | 0.6 (-5.4 – 6.6) | 0.84 | Gut/immune activation  (n=19) | 7.8 (2.7 – 12.9) | 0.003 | 6.89 (1.9 – 11.9) | 0.01 |
|  | Neurovascular  (n=27) | 0.8 (-4.8 – 6.4) | 0.78 | 4.1 (-1.1 – 9.3) | 0.12 | Neurovascular  (n=92) | 3.8 (0.8 – 6.8) | 0.01 | 3.48 (0.6 – 6.4) | 0.02 |
|  |  | **Crude (n=94)** | | **Adjusted (n=94)** | |  | **Crude (n=218)** | | **Adjusted (n=218)** | |
| Model 2: without  Postcode data | *Reference*  *(n=47)* | *Ref* | *-* | *-* | *-* | *Reference (n=99)* | *Ref* | *-* | *-* | *-* |
|  | Gut/immune activation  (n=17) | -2.6 (-8.8 – 3.6) | 0.41 | 0.1 (-6.2 – 6.4) | 0.98 | Gut/immune activation  (n=19) | 6.4 (2.0– 10.8) | 0.004 | 6.45 (2.2 – 10.7) | 0.003 |
|  | Neurovascular  (n=30) | 2.0 (-3.1 – 7.1) | 0.44 | 4.7 (-0.5 – 9.9) | 0.08 | Neurovascular  (n=100) | 2.9 (0.4– 5.4) | 0.02 | 3.09 (0.7 – 5.5) | 0.01 |

*Differences in the median CVD risk relative to the reference cluster are reported above. A higher positive (absolute) difference is indicative of a greater median risk

**Supplementary Table 5.** Sensitivity analysis that excluded individuals with prior CVD event at baseline

| **Sensitivity analysis 2 – No prior CVD event** | | | | | | | | | | |
| --- | --- | --- | --- | --- | --- | --- | --- | --- | --- | --- |
|  | **HIV-negative cohort**  **(n=62)** | | | |  | | **HIV- positive cohort**  **(n=115)** | | | |
| **Cluster** | **Crude** | | **Adjusted** | | **Cluster** | | **Crude** | | **Adjusted** | |
|  | Estimated impact on median | p-value | Estimated impact on median | p-value |  | | Estimated impact on median | p-value | Estimated impact on median | p-value |
| ***Framingham risk score (FRS)*** | | | | | | | | | | |
| *Reference* | *Ref* | *-* | *-* | *-* | | *Reference* | *Ref* | *-* | *-* | *-* |
| Gut/immune activation | 2.36 (-6.0 – 10.72) | 0.58 | 5.08 (-3.24 - 13.40) | 0.23 | | Gut/immune activation | 4.78 (-2.10 – 11.66) | 0.17 | 6.09 (-0.03 – 12.18) | 0.05 |
| Neurovascular | 1.55 (-5.84 – 8.93) | 0.68 | 1.34 (-6.66 – 9.35) | 0.74 | | Neurovascular | 2.48 (-2.02 - 6.99) | 0.28 | 1.05 (-2.99 – 5.09) | 0.61 |
| ***QRISK2*** ***score*** | | | | | | | | | | |
| *Reference* | *Ref* | *-* | *-* | *-* | | *Reference* | *Ref* | *-* | *-* | *-* |
| Gut/immune activation | 0.1 (-7.88 – 8.08) | 0.98 | 0.1 (-7.64 – 7.84) | 0.98 | | Gut/immune activation | 7.4 (1.92 – 12.88) | 0.01 | 6.25 (1.54 – 10.96) | 0.01 |
| Neurovascular | 0.9 (-6.15 – 7.95) | 0.80 | 0.9 (-5.92 – 7.72) | 0.79 | | Neurovascular | 2.8 (-0.78 – 6.39) | 0.13 | 1.69 (-1.39 – 4.77) | 0.28 |
| ***D:A:D score*** | | | | | | | | | | |
|  | *-* | *-* | *-* | *-* | | *Reference* | *Ref* | *-* | *-* | *-* |
|  | *-* | *-* | *-* | *-* | | Gut/immune activation | 8.04 (2.12 – 13.96) | 0.001 | 3.01 (-2.07 – 8.08) | 0.24 |
|  | *-* | *-* | *-* | *-* | | Neurovascular | 2.95 (-0.92 – 6.82) | 0.134 | 0.68 (-2.69 – 4.05) | 0.69 |

**Supplementary Table 6.** Sensitivity analysis that excluded statin use from adjusted regression models

|  | **People without HIV (n=94)** | | | | | **People with HIV (n=218)** | | | | |
| --- | --- | --- | --- | --- | --- | --- | --- | --- | --- | --- |
|  | **Adjusted model with  statin use** | | | **Adjusted model without  statin use** | | **Adjusted model with  statin use** | | | **Adjusted model without  statin use** | |
| *Cluster* | Estimated impact on median | p-value | Estimated impact on median | | p-value | Estimated impact on median | p-value | Estimated impact on median | | p-value |
| ***Framingham risk score (FRS)*** | | | | | | | | | | |
| Reference | *-* | *-* | *-* | | *-* | *-* | *-* | - | | - |
| Gut/immune activation | 2.74 (-4.33 – 9.82) | 0.44 | 2.71 (-4.52 – 9.95) | | 0.46 | 5.84 (1.01 – 10.67) | 0.02* | 5.50 (0.58 – 10.42) | | 0.03 |
| Neurovascular | 1.11 (-4.88 – 7.10) | 0.72 | 3.29 (-2.82 – 9.40) | | 0.29 | 3.08 (0.34 – 5.81) | 0.03* | 2.80 (0.002-5.61) | | 0.05 |
| ***QRISK2*** ***score*** | | | | | | | |  | |  |
| Reference | *-* | *-* | - | | - | *-* | *-* | *-* | | *-* |
| Gut/immune activation | 0.1 (-6.23 – 6.43) | 0.98 | -2.6 (-8.80 – 3.60) | | 0.41 | 6.45 (2.21 – 10.69) | 0.003** | 6.90 (2.31-11.48) | | 0.003 |
| Neurovascular | 4.7 (-0.54 – 9.94) | 0.08 | 2.0 (-3.12 – 7.12) | | 0.44 | 3.09 (0.72 – 5.46) | 0.01** | 3.45 (0.86 – 6.02) | | 0.01 |
| ***D:A:D score*** | | | | | | | |  | |  |
| Reference | *-* | *-* | - | | - | *-* | *-* | - | | - |
| Gut/immune activation | *-* | *-* | - | | - | 5.40 (0.66 – 10.15) | 0.03* | 5.20 (0.73-9.66) | | 0.02 |
| Neurovascular | *-* | *-* | - | | - | 1.72 (-0.99 – 4.42) | 0.21 | 2.17 (-0.39-4.72) | | 0.10 |

**Supplementary Table 7.** Comparison between demographic, lifestyle and clinical characteristics between included (n=312) and excluded (n=153) cohorts

| **Characteristic** | **Included cohort  (n= 312)** | **Excluded cohort  (n= 153)** | **p^†^** |
| --- | --- | --- | --- |
| ***Cluster*** |  |  | 0.09 |
| I – Reference | 146 (46.8) | 63 (41.2) |  |
| II – Gut/immune activation | 36 (11.5) | 11 (7.2) |  |
| III – Neurovascular | 130 (41.7) | 79 (51.6) |  |
| ***Sociodemographic*** |  |  |  |
| **Age, y, median (IQR)** | **55 (51 - 60)** | **53 (41 – 60)** | **0.001** |
| Male | 258 (82.3) | 116 (75.8) | 0.08 |
| **Race** |  |  | **0.001** |
| White | 285 (91.4) | 123 (80.4) |  |
| Black African | 27 (8.7) | 30 (19.6) |  |
| ***Cardiovascular risk factors*** |  |  |  |
| Statin use | 58 (18.6) | 26 (17.0) | 0.67 |
| Diabetes mellitus | 65 (20.8) | 29 (19.0) | 0.64 |
| Systolic blood pressure | 126 (116 - 140) | 128 (119 – 138) | 0.30 |
| Body mass index, kg/m2, median (IQR) | 25.6 (23.1 - 28.4) | 25.3 (22.9 – 29.0) | 0.99 |
| Smoking |  |  | 0.66 |
| Never | 130 (41.7) | 63 (41.5) |  |
| Former | 106 (34.0) | 57 (37.5) |  |
| Current | 76 (24.4) | 32 (21.1) |  |
| ***HIV-related factors*** | **HIV+ cohort (n=218)** | **HIV+ cohort**  **(n=125)** |  |
| **Years since HIV diagnosis, median (IQR)** | **16.1 (8.2 – 21.9)** | **12.6 (6.9 - 18.9)** | **0.03** |
| CD4 count, cells/μL, median (IQR) | 607.5 (468 – 756) | 620 (481 – 847) | 0.19 |
| **Nadir CD4 count, cells/μL, median (IQR)** | **180 (99 - 280)** | **231 (99 – 386)** | **0.03** |
| HIV RNA undetectable (<50 copies/mL) | 204 (93.6) | 112 (73.2) | 0.17 |
| **ART duration, y, median (IQR)** | **10.3 (5.4 - 17.1)** | **7.8 (3.3 – 15.7)** | **0.02** |

^†^p-value for between-cohort differences; calculated using chi^2^ (categorical variables) or Kruskal-Wallis (continuous variables)
